# From Contraception to Calculus: An Unusual Case of Intrauterine Contraceptive Device (IUCD) Migration to the Bladder, Recovering Nine Years Post-insertion

**DOI:** 10.7759/cureus.57582

**Published:** 2024-04-04

**Authors:** Zara Arshad, Anum Saleem, Muhammad Samsoor Zarak, Awranoos Ahadi, Qurratulain Umar, Rubia Afshan

**Affiliations:** 1 Research, Global Remote Research Scholars Program, Chicago, USA; 2 Internal Medicine, Shifa International Hospital Islamabad, Islamabad, PAK; 3 Internal Medicine, Bolan University of Medical and Health Sciences, Quetta, PAK; 4 Internal Medicine, Bolan Medical College Quetta, Quetta, PAK; 5 Internal Medicine, Northwest Medical Center, Tucson, USA; 6 Internal Medicine, Lahore General Hospital, Lahore, PAK; 7 Obstetrics and Gynaecology, Shaikh Khalifa Bin Zayed Al Nahyan Medical & Dental College, Quetta, PAK

**Keywords:** urinary bladder perforation, intravesical migration, calcified iucd, urinary bladder calculus, intrauterine contraceptive device

## Abstract

Uterine perforations caused by intrauterine contraceptive devices (IUCDs) have been rarely documented in medical literature. However, the migration of these devices into the bladder (intravesical migration), resulting in calculus formation, is an exceptionally uncommon occurrence. When intravesical migration happens, the IUCD may be found lodged in the bladder. In this particular case, the presence of the IUCD was detected within the bladder in the form of calculus, notably without adhering or embedding in the bladder walls. Despite being inserted nine years prior, the patient underwent seven normal deliveries without complications and remained asymptomatic concerning urinary issues until the last two years before presentation. The calculus was successfully removed from the urinary bladder via a laparotomy performed by gynecologists. This case underscores the essential role of radiological investigations and regular follow-ups in patients who report conception after IUCD insertion, as they aid in confirming the potential migration of the device and facilitate timely intervention for removal.

## Introduction

Perforations of the uterus by intrauterine contraceptive devices (IUCDs) have been reported at a rate of 0.87 in 1,000 patients; however, perforation into the bladder is a very rare occurrence [1,2]. While in the majority of cases involving urinary bladder perforation, the IUCD is typically found adhered to the bladder walls, forming calculi [3], in this particular instance, the calcified IUCD was discovered floating freely within the bladder during surgical intervention. Among treatment options, laparotomy is widely considered for the removal of IUCDs with secondary stone formation from the bladder [4]. The same was used in this case since laparoscopic or cystoscopic removal, the alternative minimally invasive methods, were unavailable in the setting.

## Case presentation

**Figure 1:**
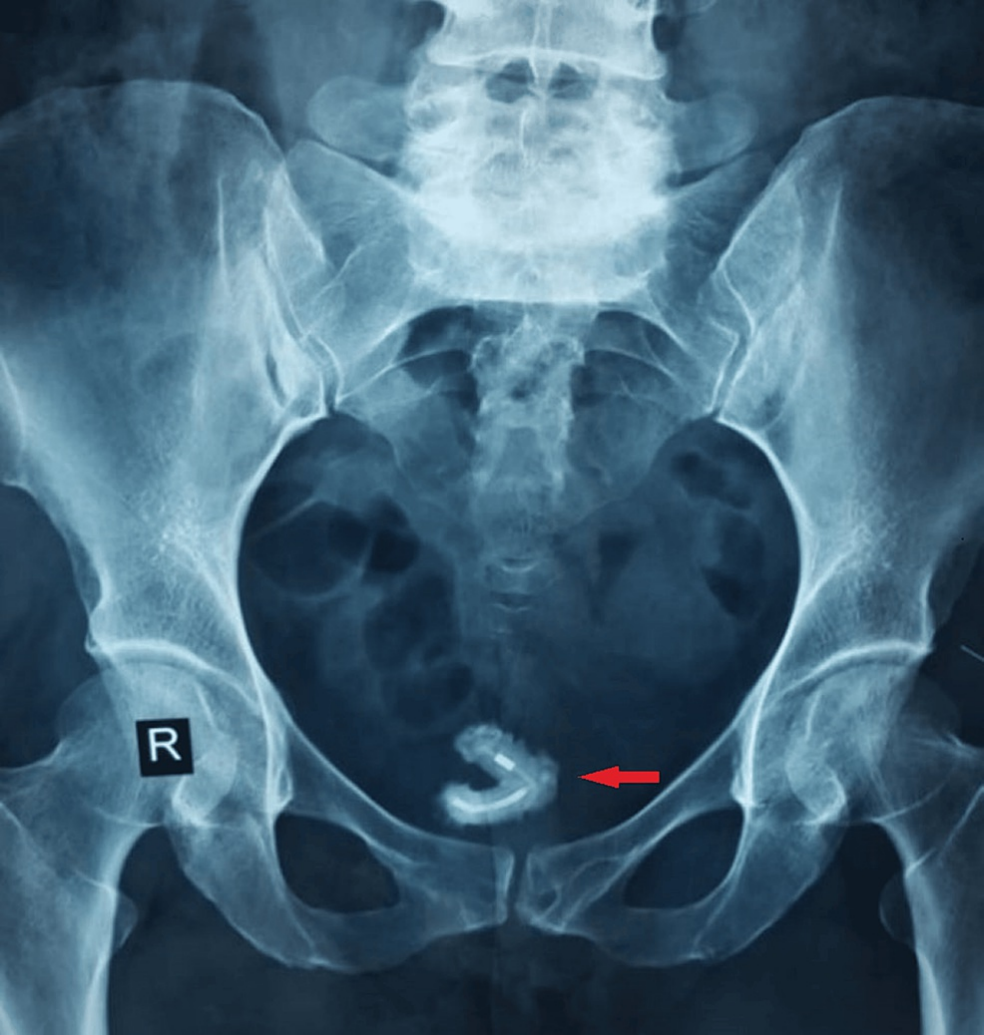
X-ray of the abdomen and pelvis X-ray of the abdomen and pelvis shows a clear V-shaped mass (old IUCD) in the urinary bladder IUCD, intrauterine contraceptive device

A 49-year-old, gravida 10 para 9, with no known comorbidities, presented to the gynecology outpatient department with a complaint of dysuria for the last 14 days. The patient complained of excessive vaginal discharge, dyspareunia, and chronic pelvic pain for the same duration. She had experienced these symptoms intermittently for approximately two years. Her history also revealed recurrent urinary tract infections for which she had been taking antibiotics previously and the insertion of an IUCD (Copper-T) nine years ago by a gynecologist. However, she had a normal delivery one year after the insertion of the IUCD. Interestingly, the patient had six further vaginal births in the subsequent eight years without complications. Her doctors suspected that the IUCD must have fallen out, but there was no radiological support for it. On her pelvic examination, tenderness was positive on deep palpation. On investigations, her routine biochemical investigations and complete blood count were normal. Her urine analysis confirmed the presence of inflammation (positive leukocytes), while the urine culture confirmed pus cells. A plain abdominal X-ray revealed a V-shaped foreign body in the pelvic region (Figure 1). On further investigation, an abdominal ultrasound confirmed a calculus within the bladder in the posterolateral region.

**Figure 2:**
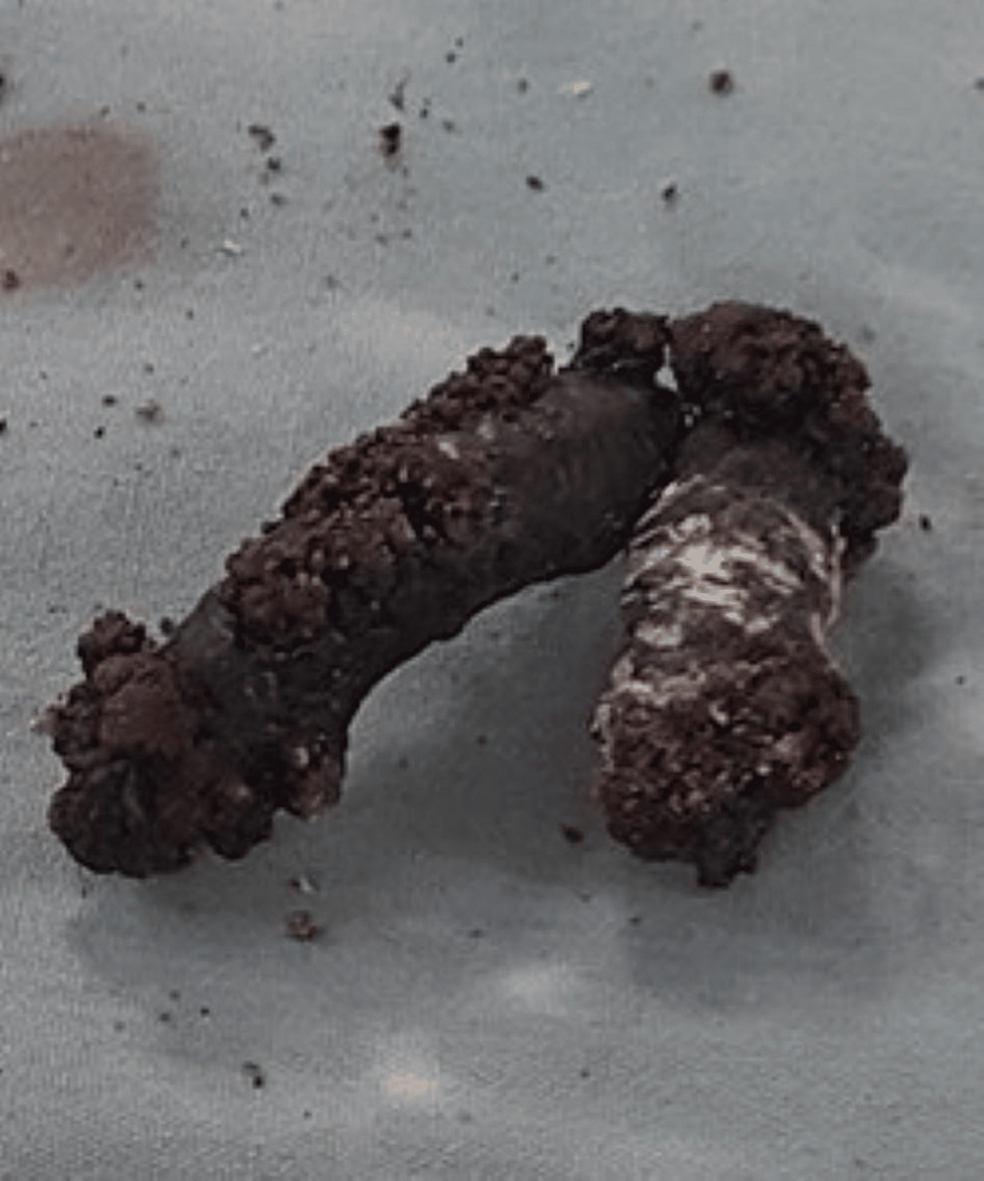
Calcified mass post-surgery Photograph of the calcified V-shaped mass recovered by gynecologists after exploratory laparotomy

Due to the unavailability of laparoscopic equipment in the facility, a laparotomy was scheduled for the removal of the calcified mass. During the laparotomy procedure, the bladder wall was observed to be normal, and there was no blood in the urine. The bladder was incised, revealing a calcified V-shaped mass freely floating in the bladder lumen, not adhering to the walls. The mass was removed with forceps in one complete piece. Upon examination, the removed mass exhibited a prominent V shape with two arms measuring 39 mm × 8 mm and 36 mm × 8 mm joined together at a junction (Figure 2). For confirmation, stone crystals were scraped, revealing an IUCD underneath. After the removal of the IUCD, no fistulous tracts were found between the wall of the bladder and the uterus. The bladder was then closed using fine, absorbable 4-0 sutures in two layers. No leakage was observed after the bladder was filled with approximately 200 mL of isotonic NaCl. A drain was inserted, and the operation was terminated. Postoperatively, the urethral Foley catheter and drain were retained for three days. No complications were observed during the postoperative period. The patient was discharged on the sixth postoperative day. At the one-month follow-up visit, she had no complaints, and her investigations were normal.

## Discussion

IUCDs are the most popular methods of reversible contraception due to their low cost, low risk, high efficacy, and low maintenance [5]. However, they are associated with rare but significant complications, including dysmenorrhea, spontaneous abortion and ectopic pregnancy, pelvic inflammatory disease (PID), menorrhagia, uterine perforations, and migration of devices to surrounding structures, which cause further complications of the structures involved [6]. Transuterine perforation of IUCDs into the abdominal cavity has been estimated at less than 0.1% in the literature; however, few have described IUCD penetration intravesically. In this case, we report the patient presenting initially with the symptoms associated with PID and urinary symptoms, which, on further investigation, revealed the presence of a urinary bladder stone, later confirmed to be a calcified IUCD.

The migration of an IUCD, coupled with the anatomical positioning of the uterus in relation to intraperitoneal structures and the bladder, creates a lethal combination. Such migrations from the uterus can happen due to uterine expulsion, displacement into an endometrial canal, or uterine perforations [7]. Subsequently, the migrated foreign body can reach adjacent organs, including the rectum, sigmoid colon, small bowel, omentum, peritoneum, retroperitoneal space, and bladder. The phenomenon and tract of transmigration of IUCD across organs are, however, quite complex and hard to comprehend [5]. Uterine perforations by IUCDs can occur either at the time of insertion or by gradual pressure necrosis of the uterine wall by the device over time. Migration of an IUCD into neighboring organs can result in complications such as bowel obstruction, peritoneal perforation, appendicitis, formation of vesical calculi, as in this case, obstructive nephropathy, fistula formation, abnormal menstrual bleeding, and intraperitoneal adhesions [8].

IUCDs that have been documented to migrate into the bladder typically result in the formation of calculi. Symptoms may appear anywhere from six months to 16 years following the insertion of the device, with some patients remaining asymptomatic [4]. Following the migration, individuals may experience urinary symptoms such as hematuria, dysuria, and increased frequency [5]. In this instance, the patient developed urinary symptoms seven years after the insertion of the IUCD, having remained asymptomatic throughout that time period.

The calculus formed within the bladder due to the presence of the IUCD typically adheres to or becomes encrusted in the bladder wall, consequently causing damage to the bladder [5,8,9]. However, in the current case, the calculus was found to be freely floating within the bladder cavity without significant damage or association with the bladder wall. Nonetheless, the patient had been experiencing urinary symptoms for two years prior to the removal of the calcified IUCD, presenting a distinct scenario concerning the intravesical migration of IUCD.

In this particular case, the diagnosis of IUCD migration was delayed until the patient presented with urinary symptoms that had persisted for several months. This situation underscores the necessity of maintaining a high level of suspicion when a pregnancy arises following a history of IUCD insertion. A plain radiograph, coupled with ultrasound, could potentially facilitate the diagnosis of migrated IUCD [9]. This case signifies the importance of regular follow-up for patients who report pregnancies following IUCD insertion.

Vaginal and pelvic examination, urine analysis, and plain abdominal X-ray reveal the pathogenicity and displacement of IUCD, including intravesical migration and calculus formation. Transvaginal and transabdominal sonographic investigations further confirm the migration of IUCD and calculus formation [8]. However, computed tomography scans and magnetic resonance imaging are more accurate means to localize misplaced IUCD [7]. In this particular case, a plain abdominal X-ray revealed calculus formation and the migration of the IUCD from the uterus into the bladder through an unknown mechanism, as there were no signs of uterine or bladder wall perforation.

The prompt removal of a migrated IUCD should be a top priority following diagnosis to mitigate the potential for complications, including chronic inflammation, infection, adhesions, and difficulties in retrieval [10,11]. Multiple methods of removal of the migrated IUCD and calculus associated with IUCD include hysteroscopy, cystoscopy, laparoscopy, and laparotomy [9]. However, cystoscopy and laparoscopy are preferred over open surgery or laparotomy due to their obvious advantages of being minimally invasive and ensuring the fewest complications [6]. Some successful innovative techniques to retrieve migrated IUDs include a combination of laparoscopy and carbon dioxide cystoscopy with partial cystectomy for the removal of partially implanted intravesical IUCD [11]. An alternative method showcased for retrieving an IUCD that had perforated the uterus and bladder involved employing a transurethral nephroscope, which reduced the risk of forming larger vesicouterine fistulas by minimizing trauma during extraction. However, the choice of approach depends on individual cases, taking into account the location and extent of migration [12]. In the present case, an open laparotomy was performed due to the unavailability of minimally invasive equipment in the facility. Post-procedure, the patient remained asymptomatic and showed an uneventful postoperative recovery.

## Conclusions

IUCDs are considered to be one of the most safe and effective methods of contraception. However, it is also associated with various complications leading to the failure of contraception. Hence, it is necessary to prevent possible failures of these devices, including misplacement and transmigration of IUCD from the uterus to adjacent organs, through regular follow-ups and awareness regarding its complications for patients. It is pertinent to educate patients with IUCDs about their potential complications and ensure regular follow-ups for proper maintenance. Suppose there is a suspicion of migration of IUCD from the uterus, radiological evidence, including X-rays and ultrasounds, of the patient, must be sought on priority. From a surgical point of view, the laparoscopic approach is the recommended approach in cases of transmigration of IUCD; however, laparotomy (an open surgical approach) should be considered for retrieval of a lost IUCD in the absence of laparoscopic equipment to minimize complications, which is also done in this case to remove the intravesically migrated IUCD.
